# Controlled Delivery of MicroRNAs into Primary Cells Using Nanostraw Technology

**DOI:** 10.1002/anbr.202000061

**Published:** 2021-03-30

**Authors:** Mara A. Pop, Benjamin D. Almquist

**Affiliations:** Department of Bioengineering, Imperial College, London SW7 2AZ, UK

**Keywords:** controlled delivery, epigenetics, microRNA, nanobiotechnology, nanostraw

## Abstract

MicroRNAs (miRNAs) are small noncoding RNAs that play key roles in post- transcriptional gene regulation. Being involved in regulating virtually all cellular processes, from proliferation and differentiation to migration and apoptosis, they have emerged as important epigenetic players. While most interest has gone into which miRNAs are involved in specific cellular processes or pathologies, the dosage-dependent effects of miRNAs remain vastly unexplored. Different doses of miRNAs can cause selective downregulation of target genes, in turn determining what signaling pathways and cellular responses are triggered. To explore this behavior, the effects of incremental miRNA dosage need to be studied; however, current delivery methods for miRNAs are unable to control how much miRNA enters a cell. Herein, an approach is presented based on a nanostrawelectroporation delivery platform that decouples the delivery from biological mechanisms (e.g., endocytosis) to enable precise control over the amount of miRNA delivered, along with demonstrating ratiometric intracellular delivery into primary dermal fibroblasts for miR-181a and miR-27a. In addition, it is shown that the nanostraw delivery platform allows efficient delivery of miRNAs into primary keratinocytes, opening new opportunities for successful miRNA delivery into this hard-to-transfect cell type.

## 1 Introduction

MicroRNAs (miRNAs) are endogenous small noncoding RNAs (20-25 bp long) involved in regulating gene expression at the posttranscriptional level.^[1]^ Also, nicknamed the “micromanagers” of gene expression due to their fine-tuning ability,^[2]^ miRNAs regulate a variety of cellular processes from differentiation and proliferation to apoptosis.^[3–5]^ They also play essential roles in conferring robustness to systems during developmental transitions when exposed to temperature fluctuations,^[6]^ as well as providing buffering to stochastic noise in cell signaling.^[7]^ Following their association with Argonaute proteins, miRNAs suppress gene expression by binding to and degrading target mRNAs or inhibiting their translation into proteins.^[8,9]^ Their vast importance in epigenetic regulation is perhaps best supported by the fact that 1) evolution has conserved miRNAs as far back in the phylogenetic tree as eumetazoans and across many diverse species,^[10]^ 2) they are highly abundant, with some miRNAs expressed as highly as 50 000 copies per cell,^[11]^ 3) the majority of protein-coding genes are miRNA targets, with over 60% of protein-coding genes containing predicted miRNA target sites,^[12]^ and 4) dysregulation of miRNAs is a common feature across a multitude of diseases,^[13]^ with a hallmark of cancer being an overall downregulation of the expression levels of miRNA,^[1]^ such as miR-15a/16-1 playing important roles in the epithelial-to-mesenchymal transition.^[14’15]^

While researchers are continually uncovering new aspects of miRNAs, such as their mechanism of action, target selectivity,^[16]^ and switch versus fine-tuning behavior,^[17]^ many times there is comparatively little effort given to other aspects of miRNAs such as understanding their dosage-dependent effects. Understanding and being able to control the dose of miRNA is crucial as the dose-dependent effects of miRNAs may lead to dual behavior in cells, likely arising from their ability to target up to hundreds of potential mRNAs with different binding affinities.^[11,18]^ One such example includes miR-17-92 that was shown to decrease cell viability of the HCT116 colon cancer cell line at low doses (0.00003 μg plasmid) but increase cell viability at high doses (0.3 μg plasmid).^[19]^

**Figure 1:**
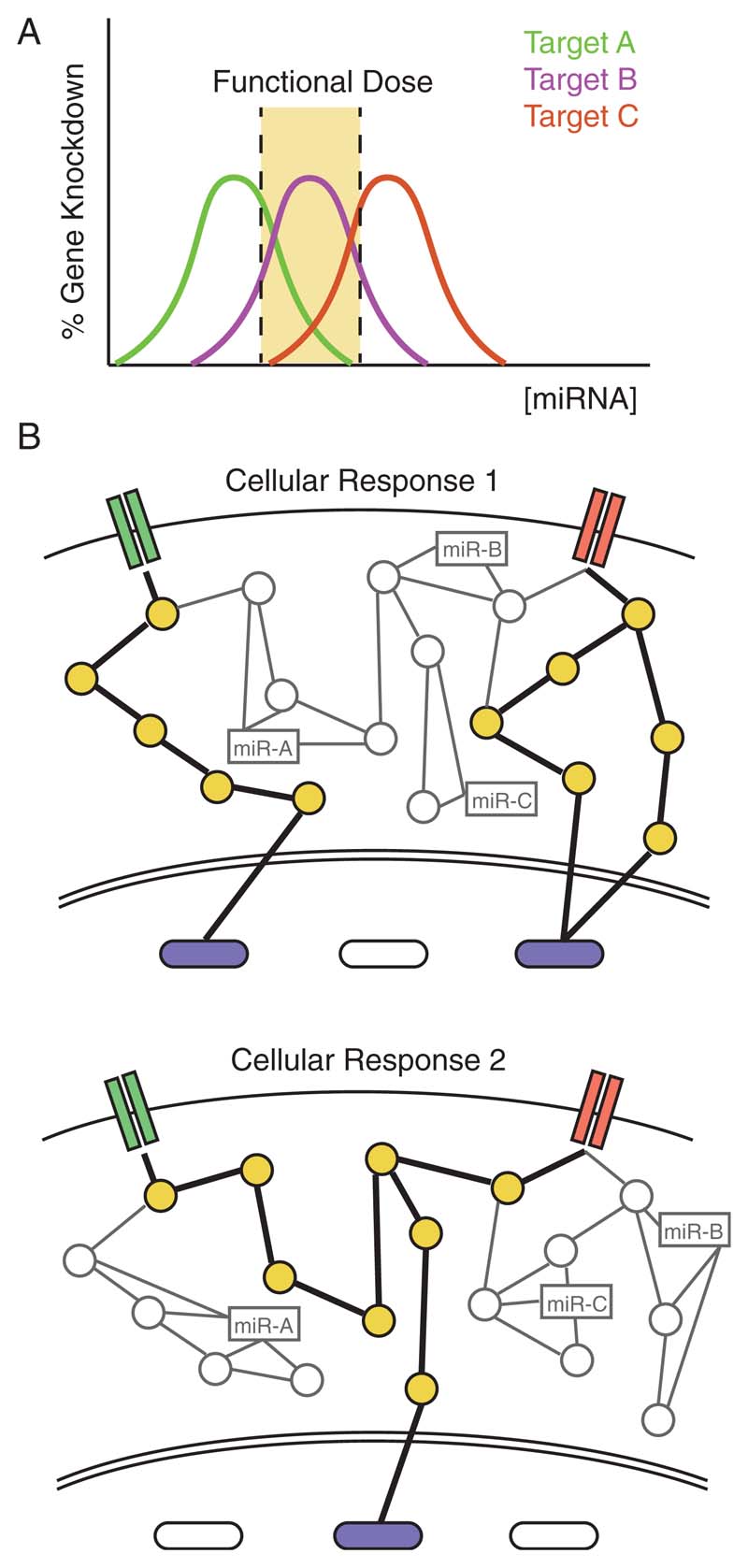
A) miRNAs have different functional doses for different target genes causing dose-dependent mRNA target selection. At a specific miRNA concentration, different target genes are suppressed by different amounts with some experiencing maximal suppression and others only minimal knockdown. The trend of gene knockdown may follow a Gaussian trend. B) Different miRNA combinations can trigger different effects at varying relative concentrations by changing the sets of genes targeted, in turn leading to different cellular behavior. This reinforces the importance of studying dosage-dependent effects of miRNAs to fully understand their behavior.

However, despite their broad implication and importance, dosage-dependent effects of miRNAs remain vastly unexplored, with most studies performed at only one concentration of miRNA. This approach provides an incomplete snapshot of a miRNA’s complex behavior as miRNAs target genes based on a narrow range of functional dose that causes maximal target suppression beyond which the particular mRNA is no longer a viable target (Figure 1A).^[19]^ This functional dose can differ for each target gene with factors such as the expression levels of the targets, the number of miRNA-binding sites on the target mRNA, and feedback loops being able to shift this narrow window for effective gene suppression.^[19]^ In addition, miRNAs were shown to repress their targets in a nonlinear manner introducing thresholds in gene expression.^[17]^

In turn, this means that for any given concentration of miRNA, different targets may be suppressed by different amounts, which can result in selective downregulation of various target genes at different doses (Figure 1B). For instance, one study showed that transfection of let-7a-7f plasmid into Huh-7 cells caused suppression of its target genes cMYC at 0.03 μg, but led to its overexpression at 0.3 μg, while for target gene CCND1 suppression occurred at 0.00003 and 0.003 μg, but not in between.^[19]^ Notably, the functional dose for cMYC required a 1000-fold larger amount for its suppression compared with CCND1, illustrating how functional doses can span several orders of magnitude for different target genes. Furthermore, as miRNAs can work cooperatively,^[1]^ this dose-dependent mRNA target selection becomes even more complicated when combinations of miRNAs, with potentially overlapping targets, are taken into account. This means that different combinations and dosages of miRNAs may start to shift target thresholds switching on or off different sets of genes, in turn activating different cell signaling pathways (Figure 1B).

Given these unique features of miRNAs, there is a need to study the incremental effects of miRNA dosage to gain a better understanding of how miRNAs regulate cellular behavior—either individually or in combinations. In turn, this requires methods that can precisely control the number of miRNAs delivered into the cells. However, due to their inherently stochastic nature,^[20]^ many current delivery methods that rely on biological mechanisms for miRNA delivery (e.g., endocytosis) are unable to precisely control how much functional miRNA is delivered. Here, we present a nanotechnology-enabled approach based on an electroporation-nanostraw (NS) delivery platform that can bypass biological mechanisms and deliver miRNAs with precise dosage control directly into primary cells. Focusing on two cell types that are important during wound healing, primary dermal fibroblasts and keratinocytes (KC), we used this nanotechnology-based approach as a tool to achieve safer, faster, and controlled delivery of miRNAs across the cell membrane, thereby overcoming various limitations of conventional delivery methods.

## 2 Results

### 2.1 Standard Delivery Techniques, Such as Liposomal-Based Delivery, Cannot Control the Dosage of Mature miRNAs Delivered

The cell membrane represents a significant barrier for delivery of biomolecular cargo, such as nucleic acids into cells. This obstacle makes it challenging to deliver RNA or DNA without the need for additional steps, giving rise to a variety of delivery methods that enable successful transfection, including viral, biochemical, and physical methods.^[21]^ A common biochemical technique is liposomal-based delivery, such as lipofectamine, that relies on the endosomal pathway for uptake of biomolecular cargo.^[22]^ In this method, the miRNA forms complexes with liposomes that are uptaken into the cells via fluid endocytosis followed by endosome formation.^[23]^ The miRNA must then escape from the endosome and associate with the RNA-Induced Silencing Complex (RISC) to yield functional miRNAs.

**Figure 2:**
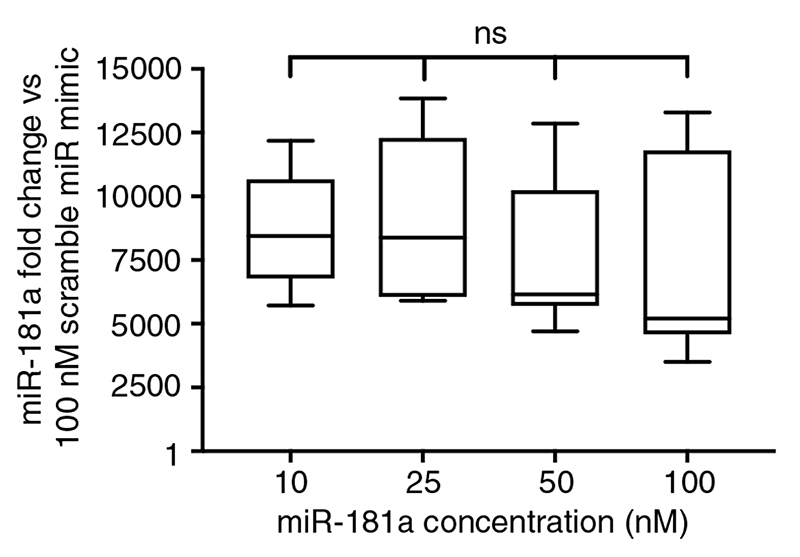
Transfection of miR-181a at 10-100 nM into primary dermal fibroblasts via RNAiMAX shows that 48 h later overexpression of miR- 181a remains similar across all concentrations of miR-181a delivered (*p* = 0.5186), thus showing that liposomal-based delivery cannot control the dosage of miRNA delivered. Box and whisker plots span min to max. Statistical analysis was performed via one-way ANOVA. *N* = 3, *n* = 9.

This method of delivery, however, is a highly random and stochastic process; an unknown fraction of miRNA nonspecifically sticks to the surface of the cells, while a second fraction fails to escape the endosomes and remains trapped in lysosomes, in turn impacting the final quantity of miRNA that successfully associates with RISC and becomes functional.^[24]^ As such, we set to determine whether lipofectamine-mediated miRNA delivery can reliably control the dosage of miRNA transfected into cells. To test this, we transfected miR-181a at 10–100 nM into primary dermal fibroblasts via lipofectamine RNAiMAX, and measured miR-181a expression 48 h posttransfection via Taqman-qPCR. We calculated the fold change in miR-181a expression relative to fibroblasts transfected with 100 nM scrambled nontargeting miRNA. Results showed that the fold change in miR-181a expression was very similar across all concentrations of miR-181a transfected, suggesting that RNAiMAX does not reliably control the miRNA dosage delivered (Figure 2). Before this, we verified that uptake of miRNA into primary fibroblasts (Figure S1A, Supporting Information) was by endocytosis (Figure S1B, Supporting Information), and using a miRNA positive control that following uptake of the miRNA, it became functional (Figure S2, Supporting Information).

Combined, these findings suggested that although RNAiMAX successfully transfects miRNA into primary dermal fibroblasts, it cannot convey robust control over how much miRNA becomes functionally available in the cytosol. This lack of control is likely at least partly due to its stochastic mechanism of delivery relying on biological mechanisms. In turn, this precludes the ability to study the dosage-dependent effects of miRNAs and suggests it is necessary to use a different approach to achieve controlled delivery of miRNAs.

### 2.2 NS-Electroporation Delivery System Can Bypass Biological Mechanisms and Achieve Effective and Dosage-Controlled Delivery of miRNAs In Vitro

To overcome the limitation posed by the stochastic liposomalbased transfection, we aimed to decouple the delivery method from relying on variable biological mechanisms by using an engineering-based approach. Specifically, we used an in vitro NS-electroporation delivery platform^[25]^ that directly injects miRNA into the cytosol via a combination of low-voltage electroporation and electrophoresis.

**Figure 3:**
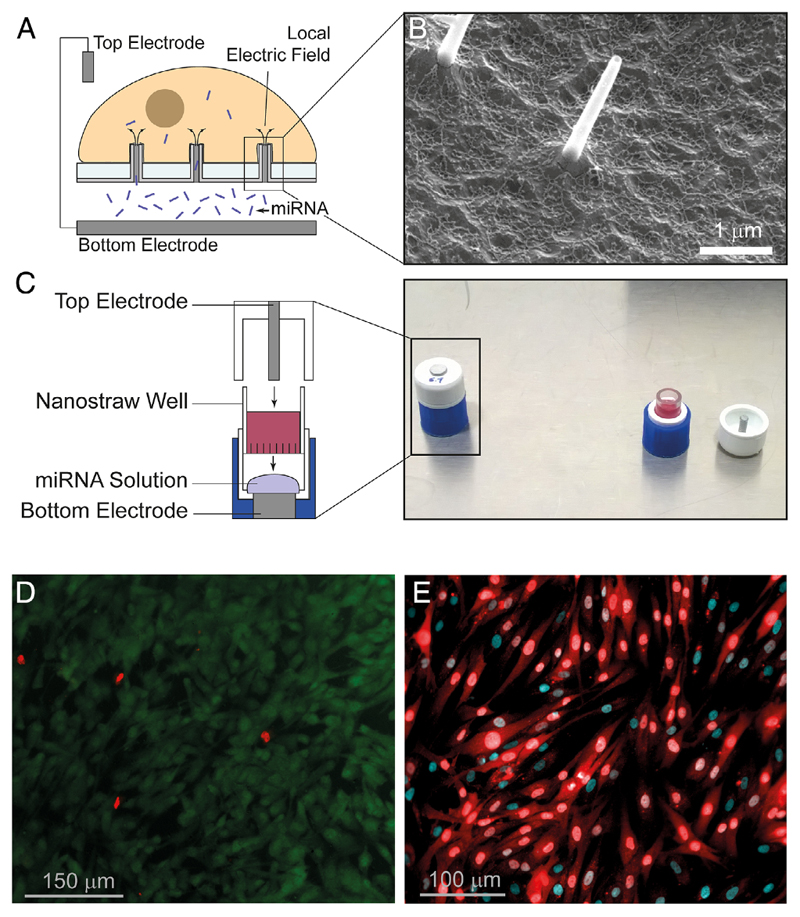
A) The tight NS-cell membrane interfaces localize the electric field at the tip of the NSs, allowing localized delivery of cargo from the underlying fluidic channel. B) Scanning electron microscope image of the NS membrane. C) Layout of the cell cap showing the cargo droplet on the bottom electrode and the NS well containing the cells. D) Primary dermal fibroblasts remain viable (green) on the NS membrane being unperturbed by the protruding NSs underneath, with very few cells dying (red). Cell viability is 92.3 ± 6.80%, *n* = 3. E) NS-mediated delivery of Cy3-tagged nontargeting miRNA (red) into primary dermal fibroblasts causes efficient and homogenous uptake into the cells’ cytoplasm 24 h after delivery (87.8 ± 3.70% efficiency of delivery, *n* = 3), thus confirming that direct intracellular access is taking place. Cyan denotes nuclear staining.

The NS platform consists of a track-etched polycarbonate membrane with protruding hollow alumina tubes (called nanostraws [NSs]) that connect to an underlying fluidic environment. Cells cultured onto the membrane “wrap” themselves tightly around the extended NS, allowing the plasma membrane to come in tight contact with them (Figure 3A). Typical NSs have a diameter of ≈100nm, 1.2-1.5 μm height (Figure 3B), and a density of 10^7^-10^8^ straws cm^-2^, which corresponds to roughly 0.1-1 straws μm^-2^. Due to the insulating properties of the polycarbonate membrane, when applying an electric field across the NS membrane (Figure S3, Supporting Information), the electric field passes through and becomes localized at the tips of the NS. This concentrated electric field induces nondamaging local pore formation in the tightly wrapped cell membrane and concurrently electrophoretically pulls up cargo from the underlying microfluidic channel through the NS into the cells.^[25]^ This allows fast uptake of biomolecules through the newly open pores, enhancing the passive diffusive process. Studies done with cell-impermeant dyes show that the pores close ≈10min after the electric field is switched off, allowing the cell membrane to heal quickly.^[25]^

In the past, this system has been characterized and shown to allow spatial, temporal, and dose control for delivery of DNA and protein,^[26]^ as well as enable co- and sequential transfection of plasmid DNA while perturbing cells minimally.^[25]^ Recently, researchers demonstrated the ability of the NS platforms to allow nondestructive longitudinal sampling of proteins and mRNA from the interior of cells.^27^ However, to date, the demonstration of dosage control has been restricted to the model HEK293 cell line, with no data regarding the ability to precisely control dosage in primary cells, nor the ability to control the delivery of miRNA.

Before using the NS-electroporation system (Figure 3C) for delivery of miRNAs into primary dermal fibroblasts, we characterized the platform extensively and verified that 1) fibroblasts maintained high viability, both on the NS membrane and after being detached from the NS membrane and recultured on plastic ware, thus confirming that the dermal fibroblasts remain unperturbed by the protruding NS underneath (Figure 3D); 2) selected 20 V and three 40 s long electroporations at 3 min intervals as the most suitable electroporation parameters for cargo delivery that maintained high cell viability (Figure S4, Supporting Information), following tests at 15-25 V and one versus three 40 s long electroporations. Previously, the delivery efficiency of DNA was shown to depend linearly with time of electroporation and quadratically with voltage,^26^ which means that a longer duration of delivery has a more significant impact on cargo uptake than small increases in the voltage applied; 3) confirmed that pores were indeed forming in the cell membrane at the selected electroporation settings as shown by efficient uptake of propidium iodide, a cell impermeable fluorescent DNA inter- calator, into the primary dermal fibroblasts following electroporation (Figure S5, Supporting Information).

Following the characterization studies of the NS-electroporation platform, we delivered fluorescently tagged nontargeting miRNA into the primary dermal fibroblasts to visualize the distribution throughout the cytoplasm. Representative fluorescence microscopy images revealed that unlike liposomal-based punctate uptake of miRNA (Figure S1B, Supporting Information), NS-mediated delivery of the fluorescent miRNA enables homogenous distribution throughout the cytoplasm 24 h postdelivery (Figure 3E) with 87.8 ± 3.70% efficacy of delivery. In turn, this confirms that the NS-electroporation system allows direct intracellular access without relying on the endosomal pathway for cargo uptake, overcoming the ambiguities that arise when using lipid-based delivery methods, and ensuring a majority of miRNA is available for incorporation into RISC.

Next, to verify that miRNAs delivered via the NS-electroporation system are functional in primary dermal fibroblasts, we tested both a miRNA positive control against GAPDH and a siRNA against CAV1 into primary dermal fibroblasts. Taqman-qPCR revealed that 48 h postdelivery, both GAPDH and CAV1 were knocked down by ≈75% (Figure S6, Supporting Information), thus confirming that NS-mediated delivery of small RNAs into fibroblasts is feasible.

**Figure 4:**
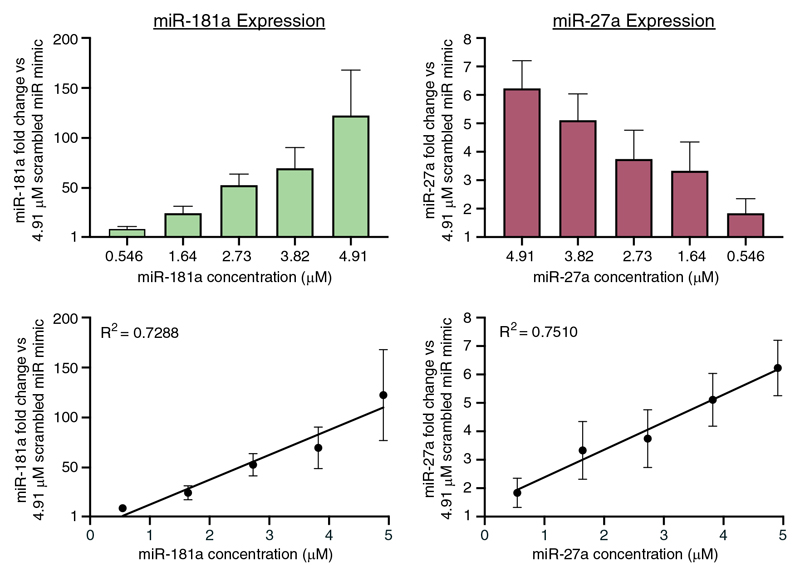
NS-mediated ratiometric and symmetrical delivery of miR-181a mimic and miR-27a mimic into primary dermal fibroblasts at 0.546-4.91 μM (0.5-4.5 μg) in the delivery buffer causes a linear increase in miRNA expression 48 h postdelivery, confirming that the NS-electroporation system can precisely control the dosage of miRNA delivered. R^2^ values were determined using linear regression analysis. *N* = 2, *n* = 8.

Following confirmation that NS can deliver functional small RNAs, we delivered mature mimics of two miRNAs, miR- 181a and miR-27a, in a ratiometric and symmetrical manner into primary dermal fibroblasts. Measurement of miRNA expression 48 h later via Taqman-qPCR revealed a linear increase in both miR-181a and miR-27a expression with increasing concentrations of miRNA in the delivery buffer (Figure 4). This pattern contrasts with liposomal-based delivery of miRNAs, illustrating that NS-mediated delivery precisely controls the dosage of miRNA, unlike the similar expression levels at all delivery concentrations when using RNAiMAX (Figure 2).

The 48 h measurement time was chosen after confirming the stability of miR mimics over a 72 h postdelivery period using a nonmammalian cell expressed control miRNA to eliminate endogenous expression artifacts. No significant decrease in miR mimic stability over time (Figure S7, Supporting Information) was detected, leading to the decision to use 48 h as the time point for measuring expression of miRNA. This time point provides the miRNA mimics enough time to exert functional effects in the cells while also providing ample time to miRNA delivered via lipofectamine (Figure 2) to escape from endosomes and become active.

**Figure 5:**
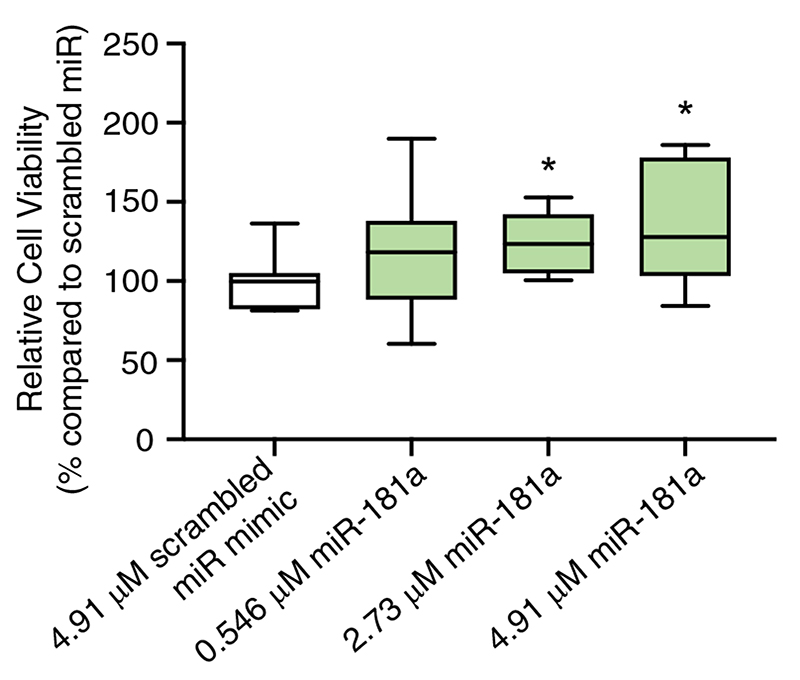
NS-mediated delivery of miR-181a mimic at 0.546-4.91 μM (0.5-4.5 μg) into primary dermal fibroblasts leads to an increase in the proliferative capacity of fibroblasts, showing a trend of increasing proliferation with increasing concentration of miR-181a. Box and whisker plots span min to max. Statistical analysis to determine significance was done via pairwise two-tailed unpaired t-tests vs scramble miR samples. *p < 0.05. *N* = 3, *n* = 6. Absolute viabilities can be found in Figure S9, Supporting Information.

After confirming the NS-electroporation system can titrate miRNA doses, we explored the impact of increasing the concentration of miR-181a on fibroblast proliferation, a miRNA shown in the past to increase proliferation.^[28]^ For this study, we delivered miR-181a mimic, spiked with scramble miR mimic, to ensure a consistent 4.9 μM total miRNA delivery concentration into primary dermal fibroblasts and measured cell viability over the ensuing 5 days via Presto Blue. Results revealed that miR- 181a exerted a proliferative effect on fibroblasts at all concentrations tested (Figure 5), with an increasing trend correlating with the increasing concentration of miR-181a in the delivery buffer. No such increasing trend in proliferation was observed when transfecting miR-181a mimic into fibroblasts at 10-100 nM via lipofectamine RNAiMAX (Figure S8, Supporting Information).

Note that the concentrations differ for these two delivery methods due to the different forms of delivery. Lipofectamine uses lipid complexation and subsequent delivery via endocytosis and is measured as a concentration in the large buffer volume within the culture well. In contrast, the NS platform has a highly concentrated solution in a small void beneath the NS well. Only a tiny fraction of this highly concentrated solution passes up into the cells via the NSs. The impact of this difference can be seen in the large difference in fold change between lipofectamine and NS delivery (Figure 2 vs 4), with the lower level of delivery via NSs likely contributing to their ability to adroitly control the dosage.

### 2.3 The NS Delivery Platform Enables Delivery of miRNAs into Hard-to-Transfect Primary KC

The physical mechanism of delivery and lack of need to rely on any biological machinery for delivery means the NS system is likely less sensitive to cell types and cell-specific characteristics, such as cell source and origin (primary vs cell line), cell density, passage and age, and cell cycle than other transfection techniques, such as viral- or biochemical-based methods.^[21]^ This ability to depend less on cell type specificity unlocks the possibility of delivering cargo into a variety of cells that are otherwise difficult to transfect using standard techniques. In the past, researchers used the NS-electroporation system to deliver mRNA successfully into hard-to-transfect cell types, such as human embryonic stem cells, primary mouse glia, and primary mouse neurons.^[26]^ Following the successful delivery of miRNAs into primary dermal fibroblasts, we tested whether the NS-electroporation system also works with primary KC, a cell type that is notoriously challenging to transfect.

Primary KC make up 90% of the cells found in the epidermis, the outermost layer of the skin. Scattered across all four to five layers of the epidermis, these cells play a significant role in providing a protective barrier against outside factors, such as pathogens, heat, or UV, and regulate water loss from the body. Also known as “the guardians of the skin,” primary KC are generally considered one of the most challenging cell types to transfect, with many common transfection methods including liposomal-, electroporation-, and viral-based methods achieving limited success.^[29]^ Low transfection efficiency may partly arise from the poor endocytotic properties of KC and high sensitivity to any sort of physical manipulation during treatments. To overcome these limitations, many studies use immortalized HaCaT cells, a widely used model of KC, due to their increased resilience, ease of propagation in culture, and improved efficiency of transfection.^[30]^ However, as with every cell line, these cells are not truly representative of their primary cell counterparts and cannot account for donor variability and unique disease traits, being unable to recapitulate the biological variation occurring in human skin.

**Figure 6:**
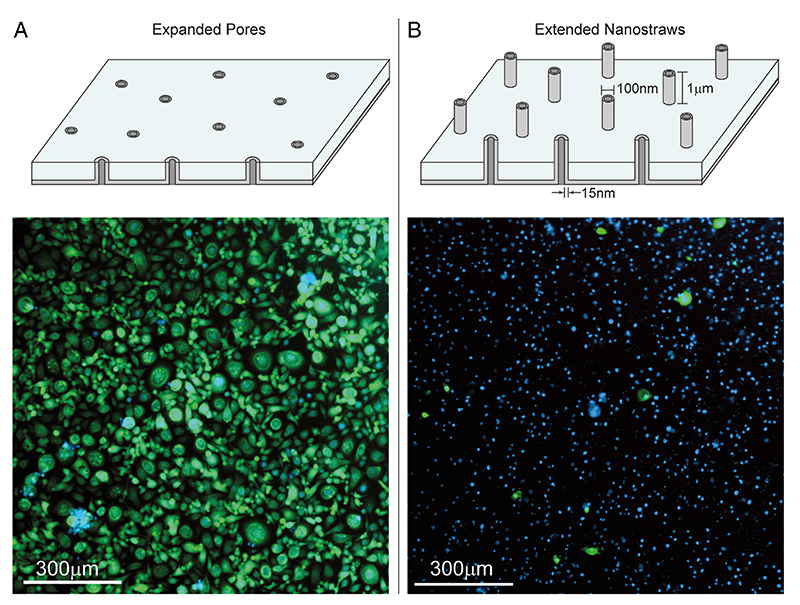
A) NS membranes with expanded pores reveal a flat surface containing pores that are not extending outwards, but have the same chemical surface modification. Primary KC seeded on this surface show high cell viability (green cells) 24 h postseeding (cell viability 97.1 ± 0.23, n = 2). B) The regular NS membrane contains extended NSs. When primary KC are seeded on this membrane, most of the cells die 24 h after seeding (cell viability 5.29 ± 4.04%, n = 2), suggesting the uneven surface created by the protruding NS is detrimentally affecting KC viability despite their nanoscale (width) and microscale (length) dimensions. Blue denotes nuclear staining.

**Figure 7:**
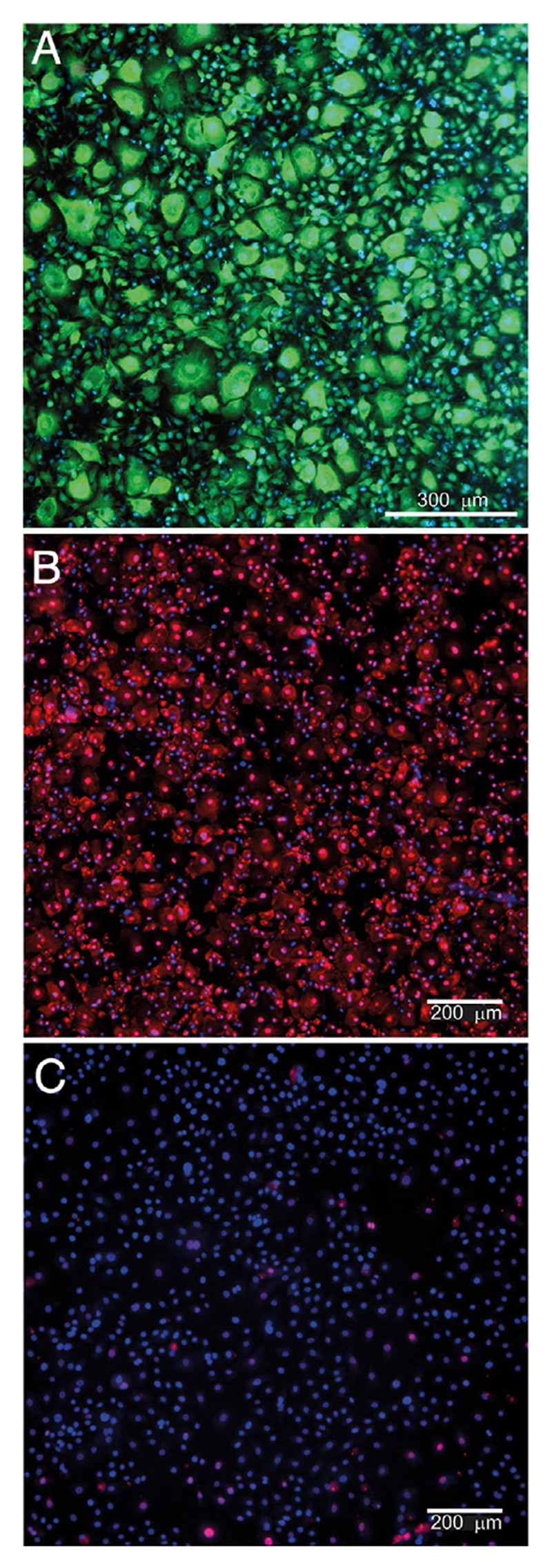
A) Precoating the NS membrane with 10% FBS maintains high viability of the primary KC on the NS membrane 24 h postseeding. Green denotes viable cells; B) NS-mediated delivery of Cy3-tagged nontargeting miRNA (red) into primary KC causes successful and homogenous uptake of miRNA 24 h postdelivery (efficiency of delivery 93.3 ± 2.57%, *n* = 3), confirming the NS-electroporation system is capable of delivering miRNAs into hard-to-transfect cells successfully; C) transfection of Cy3- tagged non-targeting miRNA (red) into primary KC via RNAiMAX causes very poor uptake of miRNA (efficiency of delivery 11.1 ± 0.46%, *n* = 3), consistent with the known difficulty to transfect primary KC with conventional methods. Blue denotes nuclear staining.

We delivered miRNAs into primary basal KC isolated from human breast tissue and subsequently expanded on fibroblast feeder layers. Initially, we used the successful delivery conditions for primary fibroblasts (see Experimental Section); however, this led to significant KC toxicity (Figure 6B). Unexpectedly, we determined that the protruding NSs themselves perturb the KC enough (Figure 6A), despite their nanoscale dimensions, to lead to cell death. This toxicity is in stark contrast to prior successful reports with other primary cells that suggest the nanoscale topography carries a negligible impact.^[27]^ To attempt to improve cell viability, we coated the NS membranes with 10% fetal bovine serum (FBS) prior to delivery and observed a significant increase in KC viability 24 h postseeding (Figure 7A), although this did not fully mitigate the negative impact on the KC, with viability starting to decline by 3 days postseeding (Figure S10, Supporting Information). However, with the coating, we observed efficient and homogenous cytoplasmic uptake of Cy3-tagged scramble miRNA (2 μM in the delivery buffer) into KC 24 h postdelivery with greater than 90% efficiency of delivery (Figure 7B). In contrast, liposomal-based delivery of the same fluorescent miRNA via RNAiMAX at 25 nM displayed significantly lower uptake of cargo 24 h postdelivery (Figure 7C), in agreement with the widely reported difficulty of transfecting primary KC. Although additional future studies are necessary, this proof-of-concept data suggests that NS-mediated delivery of small RNAs into hard-to-transfect primary KC is likely feasible with an optimized surface coating.

## 3 Discussion

MiRNAs regulate gene expression at the posttranscriptional level. These short noncoding RNA molecules become expressed in a spatiotemporal pattern to fine-tune a myriad of signaling pathways driving cellular processes, including proliferation, migration, differentiation, and apoptosis. Normal levels of miRNAs are essential for maintaining homeostasis, such that the onset and progression of a variety of diseases correlate with the dysregulation of miRNA.^[7]^ Yet, despite being common knowledge that miRNAs are essential epigenetic players, the complexity of miRNA-mediated gene regulation remains a formidable challenge. The multitude of target genes of each miRNA,^[8]^ nonlinear regulation of protein levels of targets,^[17]^ miRNA dosage-dependent effects,^[19]^ as well as cooperation with and redundancy among different miRNAs^[31]^ significantly increase the complexity of ascertaining a representative picture of the influences a miRNA may have. For instance, picking only one miRNA in a study to focus on, usually a differentially expressed miRNA in a disease, leaves a largely unexplored aspect to the situation as miRNAs do not work in isolation. Furthermore, picking only one concentration of miRNA in a study may fail to take into account the dosage-dependent effects of miRNAs.

Different doses of miRNAs are like pressing different keys on a piano, where each key can produce a diverse sound. Overexpressing or inhibiting a miRNA of interest at a specific concentration in “gain-of-function” and “loss-of-function” experiments provides only a snapshot of the miRNA’s behavior at that particular concentration but fails to take into account what happens at the other concentrations. Yet, research on dosagedependent effects of miRNAs remains limited. One reason for this might be that current delivery methods are unable to reproducibly control miRNA dosages as they rely on biological mechanisms, such as endocytosis, which is primarily a stochastic process. Such mechanisms further depend on a multitude of other factors, including cell type, the origin of cells (primary vs cell line), and the age and state of the cell (dividing vs nondividing), which can make delivery of miRNAs challenging, as is the case with primary KC. Other physical systems, such as bulk electroporation, cause inhomogeneities in the electric field around the cells, producing an uneven distribution in the pore size created in the cell membrane.^[32]^ Cells with large holes uptake large amounts of cargo or die, while cells with very small holes may have very little or no uptake. This inconsistency, in turn, makes it challenging to offer control over the delivery process and how much miRNA gets uptaken into the cells.

Here, we show that liposomal-based delivery, while resulting in efficient miRNA delivery in primary dermal fibroblasts, cannot easily control the miRNA dosage delivered in the 10-100 nM concentration range. This concentration range corresponds to the manufacturer’s suggestion of experimental concentrations with miRNAs, as well as an extensive literature search that showed that this is one of the most common ranges of miRNA to use for gain-of-function studies. Although it may be that concentrations lower than 10 nM might lead to dosage-dependent effects, we believe this is unlikely as in the past we observed that 10 nM of miRNA was the minimum threshold required for miRNA to exert functional effects in vitro. As such, we are confident that the range of miRNA dosage chosen in our study, spanning one order of magnitude, is appropriate for illustrating the limitation posed by RNAiMAX in controlling the dosage of miRNA delivered for a commonly used concentration range.

Although no other identical studies exist regarding the ability of RNAiMAX to achieve controlled delivery of mature miRNAs into primary cells, it is worth mentioning that some limited reports show control over the dosage delivered using liposomes. For instance, one study suggested that transfection of miR-22 into the WM239A metastatic melanoma cell line leads to a linear increase in intracellular miR-22 levels 48 h posttransfection.^[33]^ In this study, however, the authors used Dharmafect 1, a liposomal-based formulation for the transfection of biomolecular cargo into cell lines, as opposed to RNAiMAX and primary cells, as in our study. This difference makes direct comparisons between these two studies difficult. Similarly, a previous study indicated that the dose-dependent transfection of miRNAs is possible in the human liver cancer cell line Huh7 via lipofectamine 2000, a liposomal-based delivery formulation specific for plasmid transfection. However, in this study the authors transfected miRNA-coding plasmids as opposed to mature miRNA.^[19]^ This might not be as useful in certain diseases like diabetes, where components of the machinery responsible for miRNA processing, such as Dicer, are impaired.^[34]^ This deficiency may impact the ability to process the miRNA gene-encoding plasmids efficiently, while also being limited for cell types where liposomal-based transfection is inefficient, such as primary KC.

One way to overcome the constraints and stochastic behavior of standard delivery techniques, such as liposomal-based or bulk electroporation, is to use nanostructures that interface with cells locally at the nanoscale.^[35]^ This strategy limits the extent of cell perturbation and improves cell health while resulting in a more controllable and reliable delivery. The NS-electroporation system described in this article relies on the tight interface between the cell membrane and the alumina NSs that concentrates the electric fields through nanochannels. This geometry, in turn, allows transient nondamaging pores to open in the cell membrane for direct and localized delivery into the cells’ interior. Furthermore, the physical mechanism of delivery and the high practicality of the platform itself enable this system to precisely control and manipulate dosages of miRNAs in a straightforward, efficient, and cell-type independent manner. This unique NS-electroporation platform opens opportunities for investigating dose-dependent effects of miRNAs in vitro, as well as successfully transfects biomolecular cargo into hard-to-transfect cells, such as primary KC.

Using this system, we showed ratiometric linear codelivery of miR-181a and miR-27a into primary dermal fibroblasts, as well as homogenous and efficient uptake of fluorescently tagged nontargeting miRNA into primary dermal fibroblasts and hard-to-transfect primary KC. In some cases, the miRNA overexpression levels achievable via NS-mediated delivery may be more physiologically relevant than liposomal-based delivery. Even in the case of miR-27a that displayed very low fold changes (<10) due to high endogenous expression levels (Figure S11, Supporting Information), this level of fold change is still sufficient to drive functional outcomes. In the past, studies have shown that miRNA fold changes as low as 3-4 are sufficient to drive the development of disease in transgenic mice.^[36]^ As such, the NS-electroporation system enables a unique way to study cellular effects following small changes in miRNA expression.

Despite the physical mechanism of delivery and successful delivery into two different primary cell types, the NS-electroporation platform still requires optimization for use with primary cells that are difficult to culture. Potential issues include the optimal NS dimensions and density to minimize perturbation, ideal surface coating to support cell survival, and favorable electrical parameters to minimize cell death but maximize delivery efficiency. In our case, following extensive experimentation, we found that precoating the NS membrane with 10% FBS before seeding the KC offered a path toward maintaining the viability of KC on the NS membrane. Further studies to develop defined surface functionality, as opposed to the uncontrolled deposition of numerous proteins from FBS, may likely unlock exciting possibilities for long-term maintenance of primary KC for transfection studies.

Overall, by using this delivery platform, we can begin to take advantage of the benefits that engineering gives us, such as high precision and low variability, and use it in a biological context that intrinsically has a lot of variation and noise. In turn, this allows us to start investigating dosage-dependent effects of individual miRNAs, and in various combinations at different relative doses, to explore how signaling pathways change in response to incremental changes in miRNAs. Beginning to decipher, experimentally, the dose-response of various miRNA will help inform computational modeling for improving the development of in silico mechanistic models of the complex role miRNAs play in regulating cellular signaling.

In addition, this delivery platform opens up exciting possibilities for longitudinal studies of miRNAs to track their biological effects over time, as well as allowing sequential delivery of miRNAs at specific doses to mimic certain biological processes, such as wound healing, that involve changes in expression levels of multiple miRNAs in a spatiotemporal manner.^[37]^ Through this technique, we can gain a more in-depth understanding of the relative doses of miRNAs needed for a combinatorial miRNA-based therapy aimed at rewiring cell signaling in diseases. Ultimately, by allowing the manipulation of miRNA levels in a controlled manner, this unique delivery platform has the potential to enable fundamental studies of how signaling changes arise in disease states due to changing concentrations of miRNAs.

## 4 Experimental Section

### Fibroblast Cell Culture

1) Dulbecco’s Modified Eagle’s Medium—high glucose (Sigma-Aldrich, D5671-500ML); 2) Fetal Bovine Serum, qualified, Brazil (Gibco, 10270106); 3) L-glutamine (200 mM) (Thermofisher, 25030024); 4) Penicillin-Streptomycin (Sigma-Aldrich, P4333-100ML); 5) Trypsin-EDTA solution (Sigma-Aldrich, T4049-100ML); 6) Dulbecco’s phosphate-buffered saline (Sigma-Aldrich, D8537-500ML)

### KC Cell Culture

1) Epilife Medium, with 60 μM calcium (Cascade Biologics, MEPI500CA); 2) Epilife Defined Growth Supplement (EDGS) (Cascade Biologics, S0125); 3) Coating Matrix Kit Protein (Cascade Biologics, R011K); 4) TryplE Express Enzyme (1 ×), no phenol red (Gibco, 12604013)

### MicroRNA Mimics (Dharmacon)

1) miRIDIAN microRNA Human hsa- miR-181a-5p—Mimic (C-300552-05-0010); 2) miRIDIAN microRNA Human hsa-miR-27a-3p—Mimic (C-300502-03-0010); 3) miRIDIAN microRNA Mimic Negative Control #1 (CN-001000-01-20); 4) miRIDIAN microRNA Mimic Transfection Control with Dy547 (CP-004500-01-05); 5) miRIDIAN microRNA Mimic Housekeeping Positive Control #2 (GAPD)—Human (CP-001000-02-05)

### MicroRNA Transfection via Liposomal-Based Delivery

1) Lipofectamine RNAiMAX Transfection Reagent (Invitrogen, 13778075); 2) Opti-MEM I Reduced Serum Medium (Gibco, 31985062); 3) 5× siRNA buffer 100 mL (Dharmacon, B-002000-UB-100); 4) Water, nuclease-free (Thermofisher Scientific, R0581)

### RNA Extraction and RT-qPCR

1) PureLink RNA Micro Scale Kit (Invitrogen, 12183016); 2) QIAshredder (50) (Qiagen, 79654); 3) 2- Mercaptoethanol (Sigma-Aldrich, M6250-10ML); 4) mirVana miRNA Isolation Kit, with phenol (Ambion, AM1560); 5) Ethanol absolute (200 Proof), Molecular Biology Grade (Fisher Scientific, 10644795); 6) Taqman MicroRNA Reverse Transcription kit (Applied Biosystems, 4366596); 7) Taqman Fast Advanced Mastermix (Applied Biosystems, 4444557); 8) Hsa-miR-181a-5p Taqman microRNA assay (ID: 000480) (Thermofisher Scientific, 4427975); 9) Hsa-miR-27a-3p Taqman microRNA assay (ID: 000408) (Thermofisher Scientific, 4427975); 10) RNU44 Human Taqman microRNA assay control miRNA (ID: 001094) (Thermofisher Scientific, 4427975); 11) Precision nanoScript2 Reverse Transcription Kit (Primerdesign, RT-NanoScript2); 12) Taqman Fast Universal PCR Master Mix (2×), no AmpErase UNG (Applied Biosystems, 4366072); 13) Reference gene assays with double-dye (Taqman style) probe for human ACTB and GAPDH genes (Primerdesign, HK-DD-hu-600); 14) Custom human real-time PCR assays with double-dye probe (Taqman style) 300rxn for CAV1 (Primerdesign, DD-hu-300)

### Proliferation Assays

Prestoblue Cell Viability Reagent (Invitrogen, A13262)

### Staining

1) Hoechst 33342, Trihydrochloride, Trigydrate (Invitrogen, H3570); 2) CellTracker Green CMFDA Dye (Invitrogen, C7025); 3) Propidium iodide —1.0mgmL^-1^ solution in water (Invitrogen, P3566); 4) Vectashield mounting medium for fluorescence (Vector Laboratories, H-1000); 5) Pierce 16% formaldehyde (w/v), methanol-free (Thermofisher Scientific, 28908)

### NS-Mediated Delivery of miRNAs

1) PBS—phosphate-buffered saline (10×) pH 7.4, RNase-free (Ambion, AM9625); 2) Nuclease-free water (not DEPC-treated) (Ambion, AM9932)

### Cell Culture

Primary dermal fibroblasts were isolated from healthy human amputated foot tissues using enzymatic digestion techniques. Collected tissues were deidentified at the source and submitted to the Imperial College Healthcare NHS Trust Tissue Bank under HTA license 12275 and subcollection RSM-BA-15-062 using consent forms approved by REC Walves (12/WA/0196). Fibroblasts were cultured in high glucose DMEM supplemented with 10% FBS, 2.5 mM L-glutamine and 100 units of penicillin and 0.1 mg streptomycin mL^-1^ and split when they reached 9095% confluency.

Primary basal KC were isolated from breast skin tissue following enzymatic digestion techniques (these cells were a gift from Dr. Claire Higgins, Department of Bioengineering). For routine subculture, the KC were cultured in Epilife Medium supplemented with EDGS (1 ×) in precoated cell culture flasks with coating matrix (1:100, 30 min at room temperature) and split when they reached 80-90% confluency.

All cells were grown in incubators maintained at 37°C in normoxic atmosphere with 5% CO2 and 95% humidity and regularly tested for mycoplasma contamination. All experiments with cells were done at passage 3-7.

### Transfection of miRNAs via Lipofectamine RNAiMAX

Primary dermal fibroblasts were seeded in 24-well plates at 18 000-25 000 cells per well in 450μL antibiotic-free DMEM supplemented with 10% FBS and 2.5 mM L-glutamine and left to adhere overnight. The next day miRNAs were transfected at 10-100 nM via lipofectamine RNAiMAX for 24-48 h following the manufacturer’s protocol. Briefly, 100 μL of opti-MEM was mixed with 6 μL of RNAiMAX, followed by mixing 100 μL of opti-MEM with 2 μL of miRNA stock solution (10-100 μM). The diluted RNAiMAX (100 μL) was subsequently mixed with the diluted miRNAs (100 μL) in 1:1 ratio and left to incubate at room temperature for 10min to allow the RNA-liposome complexes to form. Following this 50 μL of miRNA- RNAiMAX mix was added dropwise to each well in triplicates and mixed well up and down to ensure homogenous distribution across the entire surface of the well.

Primary basal KC were seeded in precoated 96-well plates at 7000-10 000 cells per well in 90 μL of Epilife supplemented with EDGS and left to adhere overnight. The following day fluorescently tagged miRNA was transfected at 25 nM via RNAiMAX for 24 h following the same protocol for transfection as for the primary fibroblasts, while the volumes of reagents used were normalized for the 96-well plate format.

### Cell Staining and Fluorescence Microscopy Imaging

Cell staining was done with nuclear dye Hoechst 33342 (7 min at room temperature in the dark), Cell Tracker Green (5 μM for 15-30 min at 37 °C), propidium Iodide (0.1 mgmL^-1^ for >10 min at room temperature), while long-term cell preservation was maintained by fixing the cells with 4% PFA (10 min at room temperature). Cells were imaged with the Inverted Widefield Zeiss Axio Observer microscope or with the Zeiss PALM MicroBeam Laser Capture Microdissection. Images were subsequently processed in Fiji.

### NS-Mediated miRNA Delivery

To deliver miRNAs into primary dermal fibroblasts, we used a prototype NS-electroporation system. The system entailed culturing cells in plastic NS wells (6.5 mm inner diameter) with a NS membrane glued to one end and NSs facing upward. The NS well was then carefully inserted into a cell cap that contained a bottom electrode capable of holding a 65 μL miRNA cargo droplet of 0.0001-0.1 × PBS and a positive electrode in the lid of the cell cap. The cell cap was placed into the electroporation device that was connected to a power supply, while electroporation was initiated when the electroporation device was switched on. As the NS membrane is porous due to the hollow NSs, electrical continuity is ensured throughout the cell cap, causing an electrical current to pass through the cell cap that can be monitored during electroporation.

Primary dermal fibroblasts were seeded at 30 000-50 000 cells per NS well in 350 μL full DMEM medium and returned to the incubator for 12 h to allow the cells to start adhering to the NS membrane. Next, the NS wells (placed in a 24-well plate) were centrifuged at 1400 rpm for 4 min to ensure the cells came in close contact with the NSs. MiRNAs were added at 0.546-5.46 μM in the delivery buffer (0.1-0.0001 × PBS) in the cargo droplet, followed by inserting the NS well with the cells in the cell cap. The cell caps were electroporated at 20 V, 40 Hz, 200 μs for 120 s over three bursts of 40 s at 3 min intervals. During electroporation the electrical current passing through the cell cap was recorded using a PicoScope to ensure no air bubbles were present in the cargo droplet that would otherwise cause electrical discontinuity in the cell cap. After electroporation the NS wells were returned to the incubator for another hour and then the fibroblasts were detached from the NS membrane using trypsin-EDTA (10 min at 37 °C) and reseeded into 24-well or 96-well plates for 24-48 h for further functional assays, i.e., RNA extraction or staining.

Primary basal KC were seeded at 50 000 cells per NS well in 350 μL in full Epilife Medium on the NS membrane that was precoated with 10% FBS or plasma treated with oxygen at 0.5 mbar for 5 min and left to adhere overnight. The following day Cy3-tagged miRNAs were delivered at 2 μM at 15 V, 40 Hz, 200 μs for 40 s in 0.0001 × PBS. Following electroporation, the medium in the NS well was replaced and the bottom of the NS membrane rinsed with PBS. The NS wells were returned to the incubator for another 24 h prior to staining and imaging.

### RNA Extraction and qPCR for miRNAs

Prior to cell lysis cells were washed with PBS three to four times or trypsinized and pelleted. RNA was extracted using the miRVana miRNA Isolation Kit that allows isolation oftotal RNA containing small RNAs. Following isolation RNA integrity was checked on 15% polyacrylamide urea gels and confirmed to be intact, while RNA purity was confirmed via Nanodrop 2000 to be high. Reverse transcription was performed on 10 ng oftotal RNA using miRNA-specific stem loop primers, part of the Taqman MicroRNA Reverse Transcription kit (Applied Biosystems), following the manufacturer’s instructions. Following formation of cDNA qPCR was run on the ABI 7500 fast machine using Taqman miRNA assays (Thermofisher Scientific) and the Taqman Fast Advanced Mastermix (Applied Biosystems) according to the manufacturer’s instructions. Changes in miRNA expression was calculated using the 2^—ΔΔCT^ method after confirming that the amplification efficiency of the small RNA assays was ≈100%. The small nucleolar RNU44 served as the internal normalization gene after confirming that its expression remained stable after transfection of miR-181a or miR- 27a (Figure S12, Supporting Information).

### RNA Extraction and qPCR for mRNAs

Prior to cell lysis cells were washed with PBS three to four times. RNA was extracted using the PureLink RNA Micro Scale Kit (Invitrogen) that isolates large RNAs. Following isolation RNA integrity was checked on 1 % native agarose gels and confirmed to be intact, while RNA purity was confirmed via Nanodrop 2000 to be high. Reverse transcription was performed on 100 ng of total RNA using oligodT primers part of the Precision nanoScript2 Reverse Transcription Kit (Primerdesign) following the manufacturer’s instructions. Following formation of cDNA qPCR was run on the ABI 7500 fast machine using double-dye Taqman probes (Primerdesign) and the Taqman Fast Universal PCR Master Mix (2×), no AmpErase UNG (Applied Biosystems) according to the manufacturer’s instructions. Changes in mRNA expression were calculated using the 2^—ΔΔCT^ method, while beta actin (ACTB) served as the internal normalization gene. The amplification efficiency of the double-dye Taqman assays was further confirmed to be ≈100%, which justified the use of the 2^—ΔΔCT^ method.

### Proliferation Assays

Following NS-mediated delivery of miR-181a according to the protocol described earlier, the fibroblasts were detached from the NS membrane and reseeded into 6-10 wells of a 96-well plate in full DMEM medium. Proliferation was measured on day 1 and day 5 via Presto Blue (1:10, 60 min incubation at 37 °C) by measuring fluorescence at 560/590 nm on the Varioskan Flash microplate reader. The fold change in proliferation over 4 days was determined and compared with cells to which 4.91 μM of scrambled miRNA was delivered.

### Graph Generation and Statistical Analysis

All graphs were plotted in GraphPad Prism. All statistical analysis was done in GraphPad Prism. For comparisons of three or more groups one-way ANOVA was used, followed by posthoc Tukey’s multiple comparisons test. For comparison of two groups unpaired two-tailed t-tests were performed. Normality of data were checked using the Shapiro-Wilk test. All experiments were done independently at least two times in at least three technical replicates per experiment unless otherwise stated. Replicate details are denoted by *N* for biologically independent samples (different biological donors), while *n* is used for independent replicates per biological donor. Statistical significance was acknowledged if *p* < 0.05.

## Supplementary Material

Supporting Information is available from the Wiley Online Library or from the author.

Supporting Information

## Data Availability

The data that support the findings of this study are available from the corresponding author upon reasonable request.
