# Supplementary material for: Controlled Delivery of MicroRNAs into Primary Cells Using Nanostraw Technology
Source: Adv Nanobiomed Res. Author manuscript; Available in PMC 2021 Jun 22. (PMC7611046; doi:10.1002/anbr.202000061)
Supplement: Supporting Information [file EMS124553-supplement-Supporting_Information.pdf]

SUPPLEMENTARY DATA

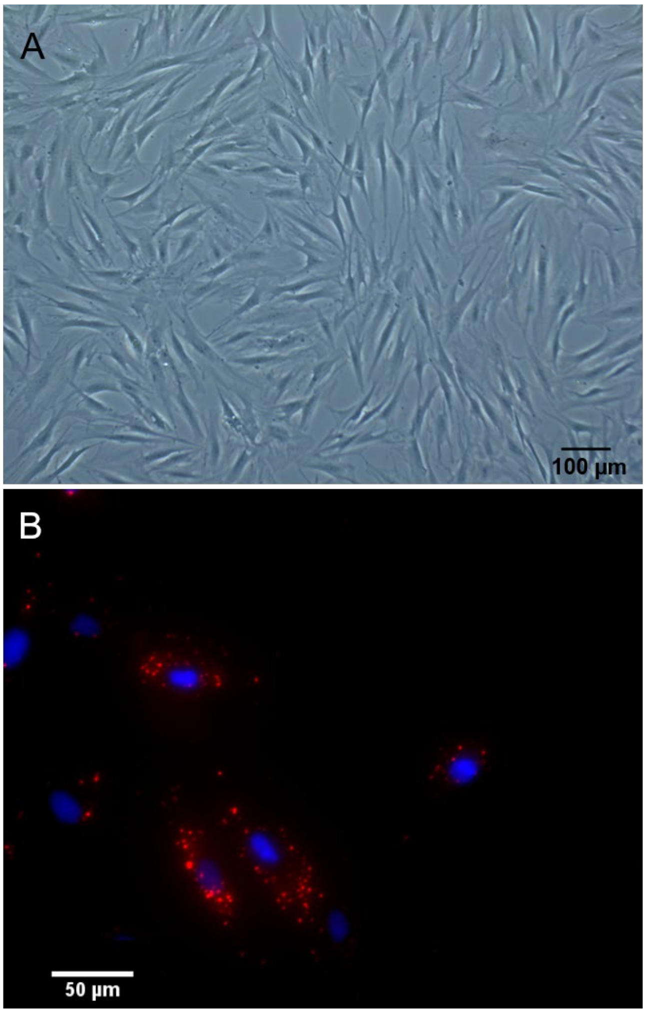

Figure S1: (A) Primary dermal fibroblasts in culture isolated from human amputated foot tissue. (B) Transfection of dy547-labelled scramble miRNA mimic (red) into primary dermal fibroblasts via RNAiMAX at 10-30 nM leads to punctate uptake of the miRNA into the cells 24h later, consistent with the activation of the endosomal pathway. Blue denotes nuclear staining.

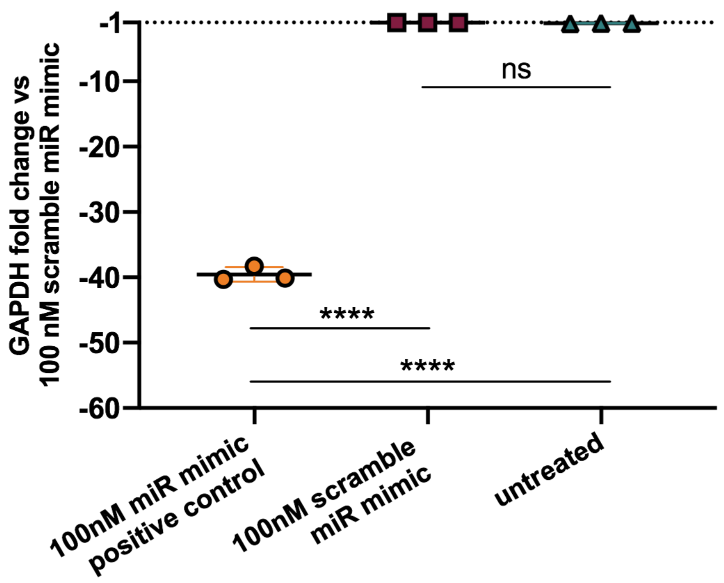

Figure S2: transfection of miRNA mimic positive control against GAPDH into primary dermal fibroblasts via RNAiMAX at 100 nM leads to effective gene knockdown compared to scramble non-targeting miR mimic or untreated cells, thus confirming that liposomal-based delivery works very efficiently at transfecting functional miRNAs into primary fibroblasts. Data points denote mean ± standard deviation. Analysis of statistical significance was performed via one-way ANOVA, followed by Tukey's multiple comparisons test. ****$p<0.0001$, $n=3$

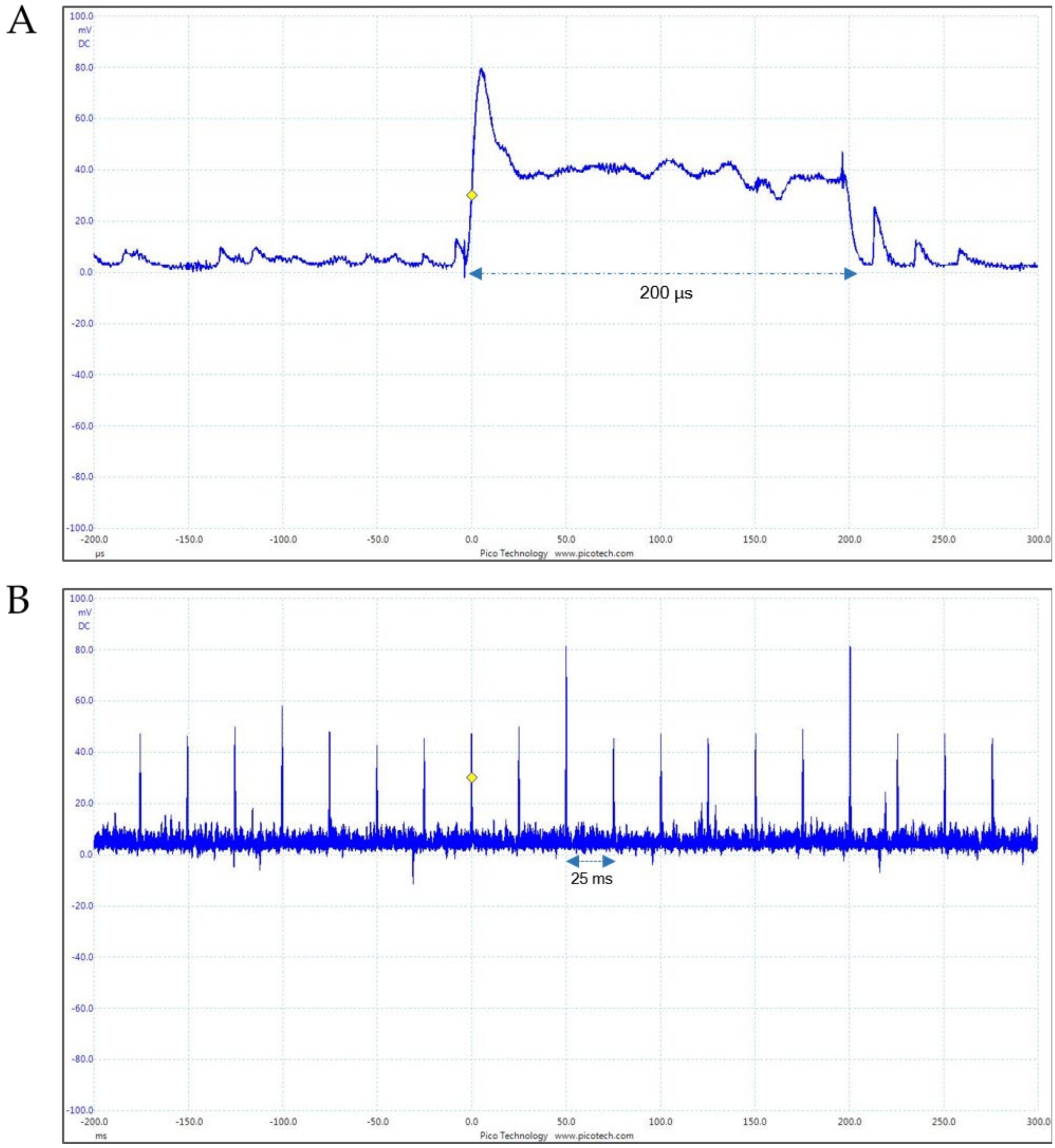

Figure S3: typical electrical signal recorded by the picoscope during NS-mediated electroporation. (A) The signal recorded has a square waveform of 200 µs pulse width and an amplitude of 40 mA. (B) This waveform was repeated every 25ms equivalent to a frequency of 40 Hz, thus resembling the selected parameters.

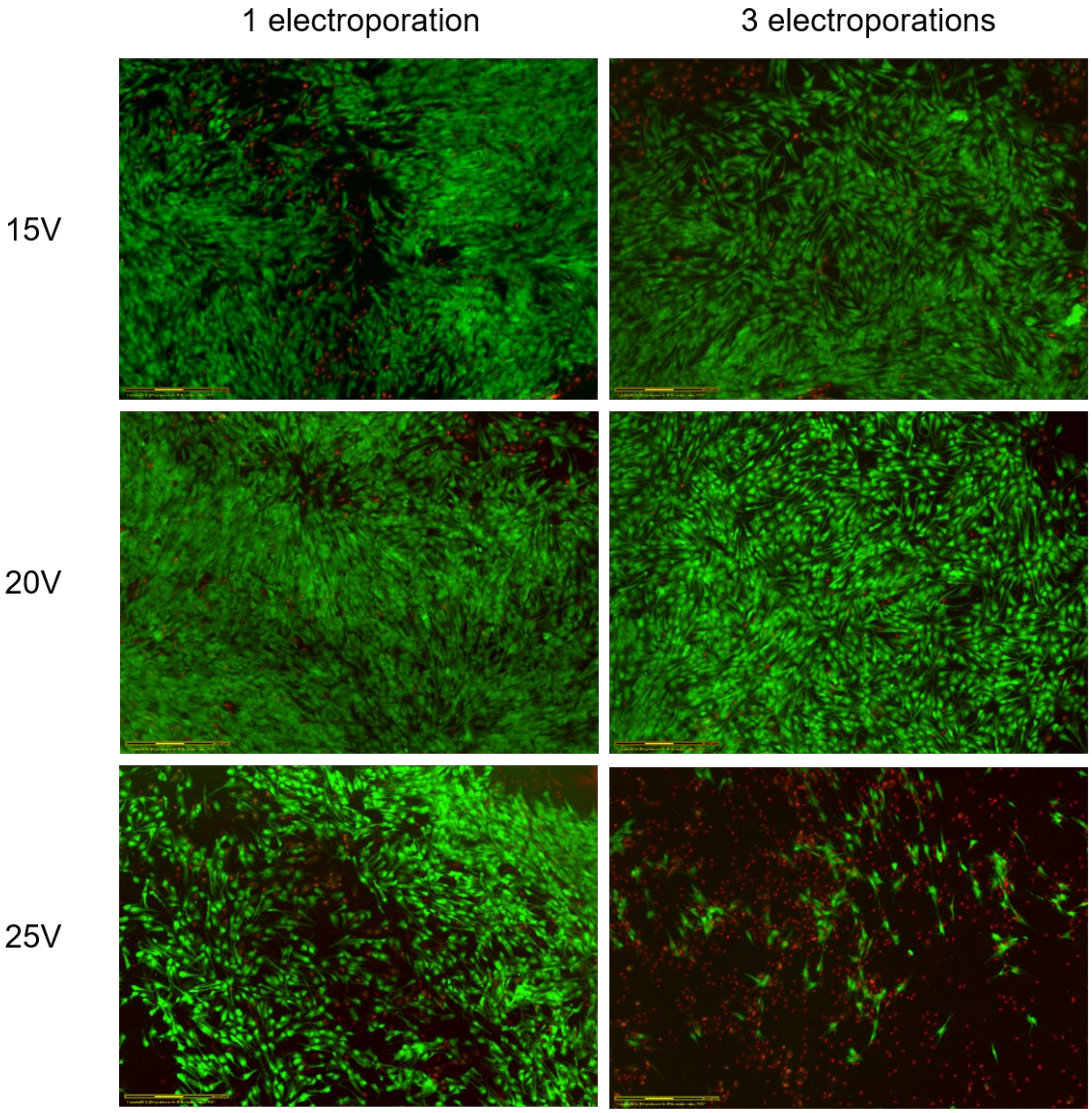

Figure S4: electroporation of primary dermal fibroblasts at 15-20V for one or three 40s long electroporations shows cells no longer maintain high cell viability at 25V, three electroporations, thus suggesting 20V is a safer electroporation parameter. Efficiency of delivery was shown in the past to depend linearly with time of electroporation, but quadratically with voltage of delivery, which means that small increases in the duration of delivery (Δt) have more significant impact on cargo uptake than small increases in the voltage applied (ΔV). As such, we decided to use 20V and three electroporations as opposed to 25V with one electroporation as the delivery parameters. Green colour denotes viable cells, red colour denotes dead cells. All scale bars denote 300 μm

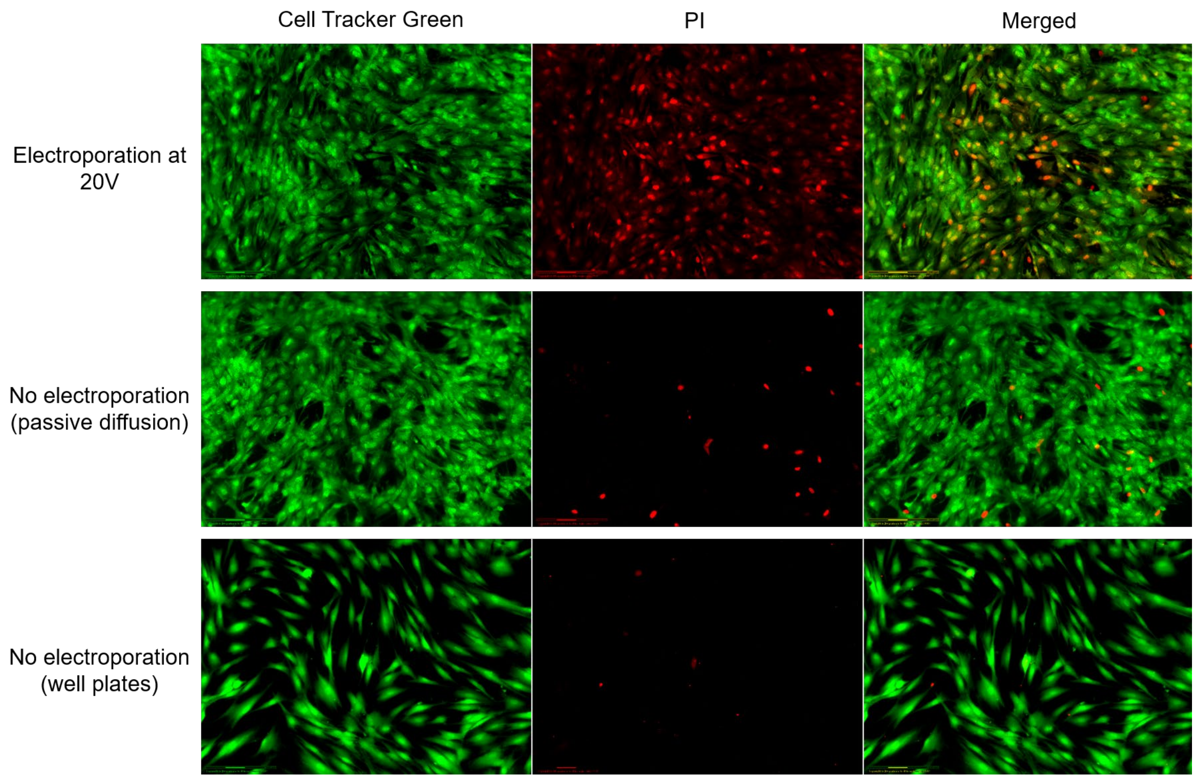

Figure S5: NS-mediated delivery of propidium iodide (PI) - a cell impermeable DNA intercalator – leads to efficient uptake into primary dermal fibroblasts (red), while maintaining high cell viability (green), thus confirming pore formation takes place in the cell membrane. In the absence of electroporation, i.e. passive diffusion very little PI gets uptaken by the cells, thus corroborating the importance of the electric field in cargo uptake. Green colour denotes viable cells – as stained via Cell Tracker Green, red colour denotes cellular uptake of PI. All scale bars denote 150 µm

A

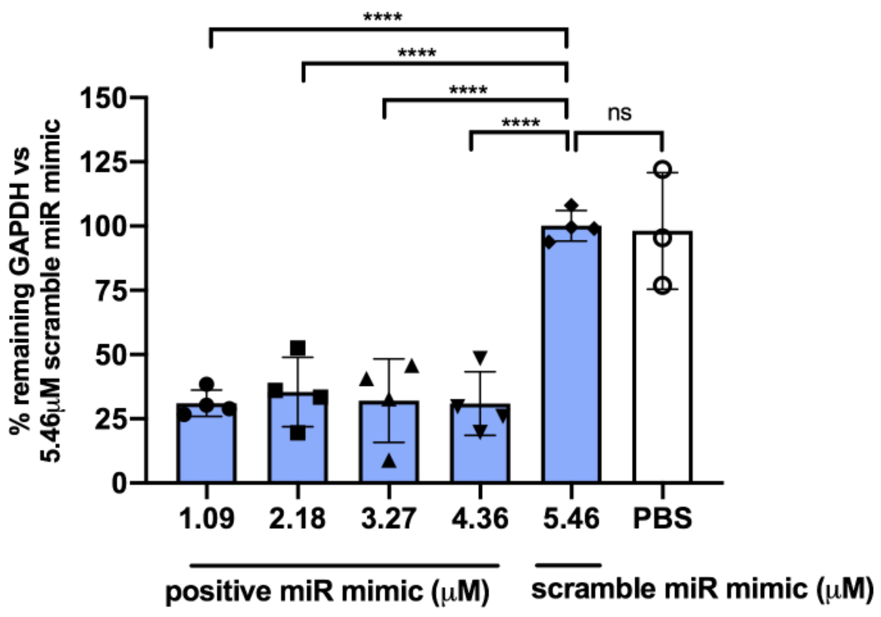

B

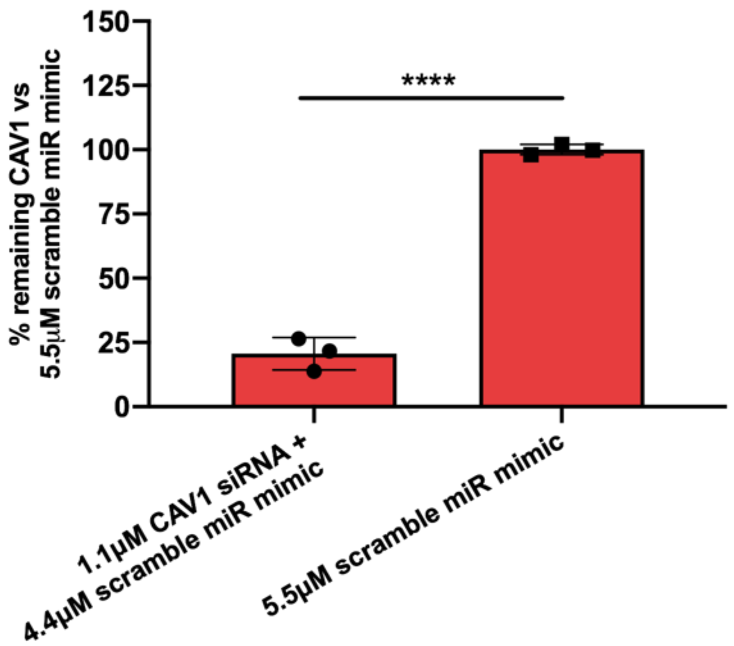

Figure S6: (A) NS-mediated delivery of miRNA mimic positive control against GAPDH into primary dermal fibroblasts shows efficient gene knockdown 48h post delivery. The large knockdown further shows the miRNA mimic positive control behaves like an siRNA rather than a fine-tuning miRNA due to chemically enhanced properties by the suppliers to increase efficacy. Bars denote mean ± standard deviation. Statistical analysis to determine significance was done via one-way ANOVA, followed by posthoc Tukey's multiple comparisons test. ****$p<0.0001$, $n=4$; (B) NS-mediated delivery of siRNA against CAV1 leads to efficient gene knockdown 48h post delivery, thus confirming the NS-electroporation system works very well at delivering functional small RNAs. Bars denote mean ± standard deviation. Statistical analysis was performed via two-tailed unpaired t-test. ****$p<0.0001$, $n=3$

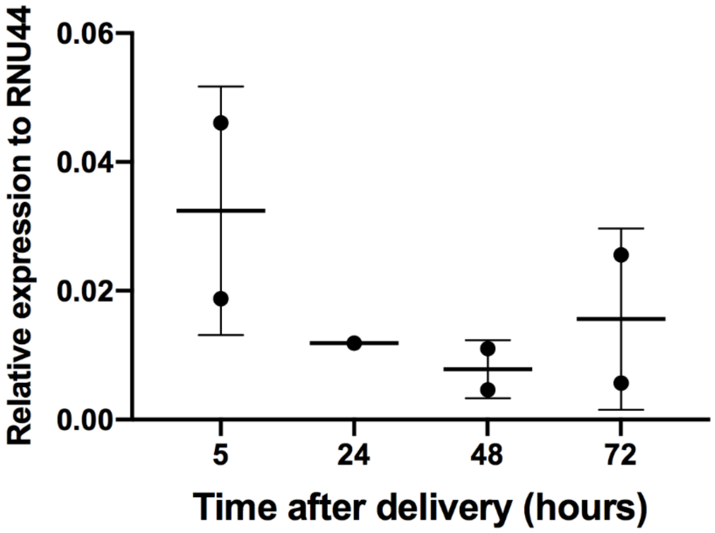

Figure S7: expression of scrambled miRNA in primary dermal fibroblasts at 5h, 24h, 48h and 72h post NS-mediated delivery of scrambled miRNA mimic shows no significant change over time. This confirmed that miRNAs remain stable in the cells over a long period of time, which led us choose 48h as the time point for measuring over-expression of miRNAs. Data denote mean ± standard deviation. Statistical analysis was performed via one-way ANOVA. p= 0.4592, n=2.

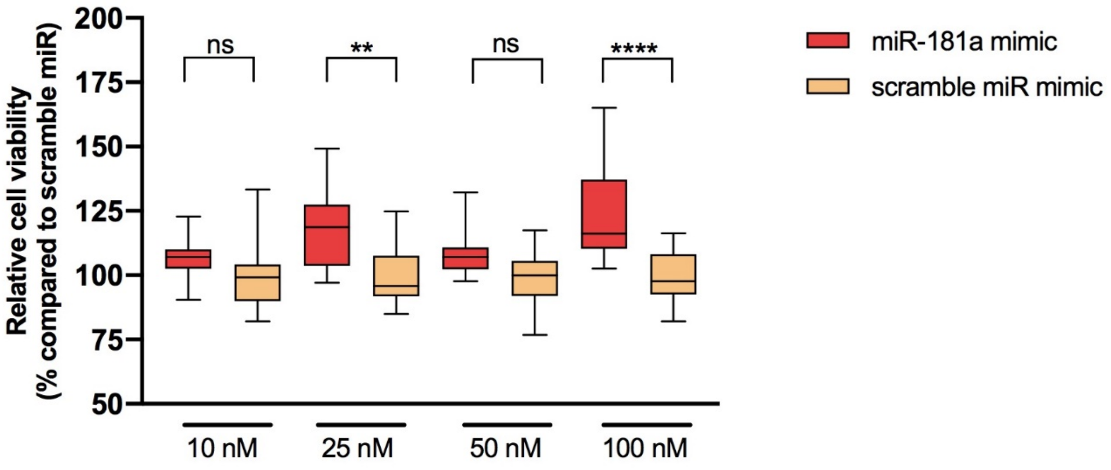

Figure S8: fibroblast proliferation goes up following transfection of miR-181a mimic at 10-100 nM via lipofectamine RNAiMAX at every concentration of miR-181a mimic tested (10nM, 25nM. 50nM, 100nM). No trend of increasing proliferation with increasing concentration of miR-181a mimic was observed. Proliferation was measured on day 5 post-transfection and cell viability was calculated relative to cells transfected with scrambled miRNA mimic. Box and whisker plots span min and max. Statistical analysis was performed via one-way ANOVA, followed by posthoc Tukey's multiple comparisons test. **p<0.01, ****p<0.0001. N=2, n=18

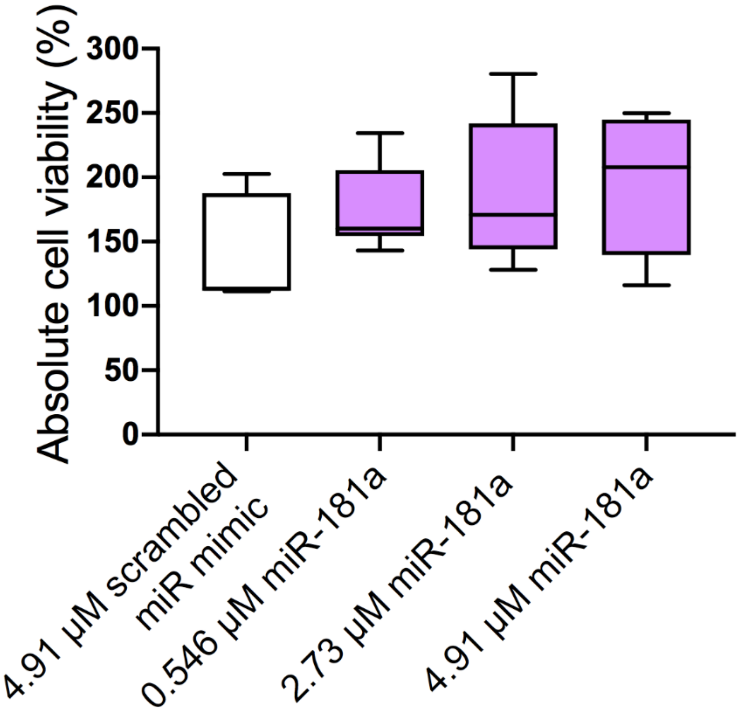

Figure S9. Absolute viabilities (unnormalized) following NS-mediated delivery of miR-181a at different dosages.

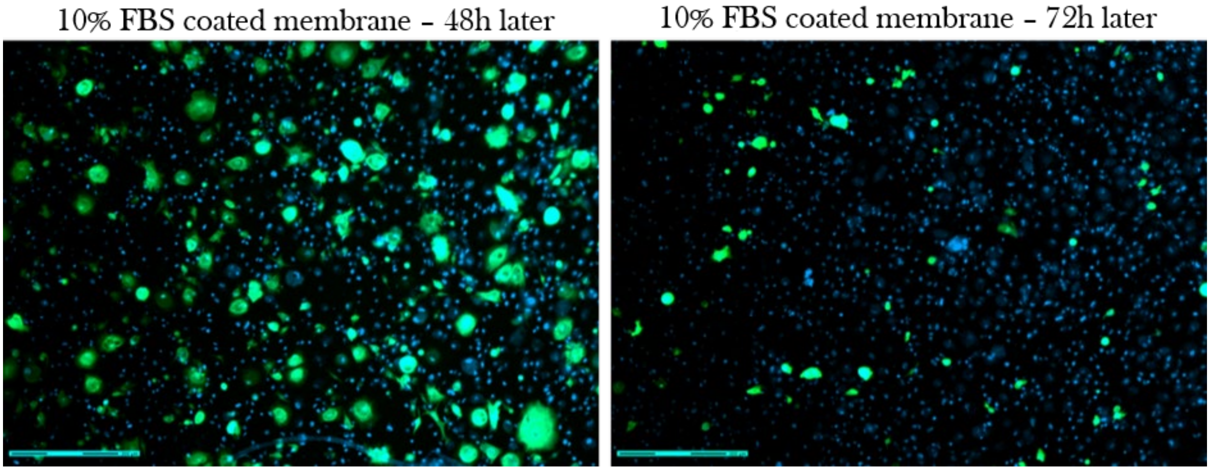

Figure S10. Primary KC seeded on 10% FBS coated NS membrane for longer than 24h start to die off, with viability decreasing significantly by day 3 post-seeding. This suggests that the positive effect of FBS is short-lived and further optimisation is needed to maintain long-term viability on the membrane. Green denotes viable cells, blue denotes nuclear staining. All scale bars denote 300 μm

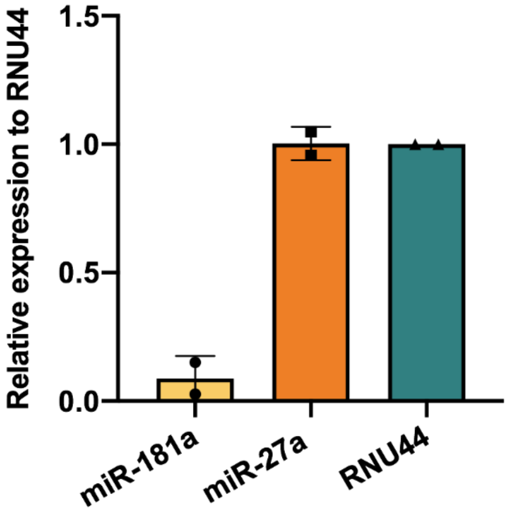

Figure S11: endogenous miR-181a is very low expressed in primary dermal fibroblasts, whereas endogenous miR-27a levels are high. The endogenous levels of the miRNAs are calculated relative to RNU44, a small nucleolar housekeeping RNA used as the internal normalisation gene. Bars denote mean ± standard deviation. n=2.

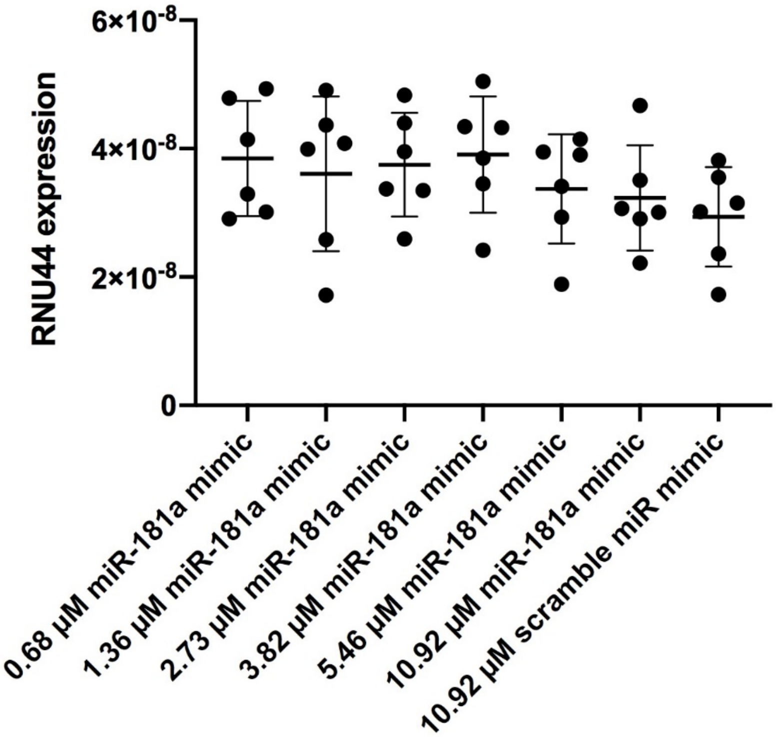

Figure S12: RNU44 is a suitable internal normalisation gene for qPCR on miRNAs. Expression of RNU44 remains stable following NS-mediated delivery of miR-181a mimic (0.68 -10.92 µM) into primary fibroblasts compared to fibroblasts containing scrambled miRNA mimic. Values are expressed as 2^{-CT}. Data denote mean ± standard deviation. Statistical analysis was performed via one-way ANOVA. $p= 0.4877$, $n=6$.
